# Coarse-Grained Simulation of Myosin-V Movement

**DOI:** 10.1155/2012/781456

**Published:** 2012-05-23

**Authors:** Zoe Katsimitsoulia, William R. Taylor

**Affiliations:** Division of Mathematical Biology, MRC National Institute for Medical Research, The Ridgeway, Mill Hill, London NW7 1AA, UK

## Abstract

We describe the development of a hierarchic modelling method applied to simulating the processive movement of the myosin-V molecular motor protein along an actin filament track. In the hierarchic model, three different levels of protein structure resolution are represented: secondary structure, domain, and protein, with the level of detail changing according to the degree of interaction among the molecules. The integrity of the system is maintained using a tree of spatially organised bounding volumes and distance constraints. Although applied to an actin-myosin system, the hierarchic framework is general enough so that it may easily be adapted to a number of other large biomolecular systems containing in the order of 100 proteins. We compared the simulation results with biophysical data, and despite the lack of atomic detail in our model, we find good agreement and can even suggest some refinements to the current model of myosin-V motion.

## 1. Introduction

### 1.1. Molecular Motors and Myosin V

Molecular motors are ubiquitous in both prokaryotes and eukaryotes and are essential to movement in all living organisms. Although structurally and functionally diverse, they all share at least one common characteristic that is an ability to convert chemical energy into mechanical work. In a single eukaryotic cell, there are at least 100 different types of molecular motors present responsible for the active transport of intracellular material across large distances within the cell. In addition to driving these important subcellular processes, molecular motors can also drive the movement of entire cells, such as lymphocytes and embryonic cells, which must also travel over great distances. The cytoskeletal filament system found in eukaryotic cells acts as a scaffold to mediate directed movement within the cell for one such group of molecular motor proteins. To achieve this, motor proteins such as myosin, kinesin, and dynein walk along the surface of polymerized actin (microfilament) and tubulin (microtubule) tracks via specific interactions with the actin and tubulin respectively. Despite variations among them, these molecular motors all appear to have converged on a core mechanism that couples ATPase activity to generate force and movement/motion via a biased conformational change.

All molecular motors that operate on actin filaments belong to the myosin superfamily. With at least 35 different classes of myosins [2], these cytoskeletal motor proteins are associated with a host of physiological roles in cell motility, including muscle contraction, chemotaxis, cytokinesis, pinocytosis, and targeted vesicle and organelle transport [3, 4]. Class V myosins, in particular, have been shown to transport cargo such as endoplasmic reticulum in neurons, melanosomes in melanocytes, and mRNA in yeast [5–7]. Small variations in enzymatic activity/kinetics and structural adaptations to an, otherwise, highly conserved catalytic motor domain allow the different myosins to generate diverse types of motility in the cell. Next to class II myosins, Myosin V is probably the most studied class and rivals myosin II as the best characterized myosin with respect to elucidating its molecular basis for motion [8].

### 1.2. Processivity and Duty Cycles

Processive motors undergo multiple chemical cycles before detaching from the filament track to which they are bound. Myosin V is a classic example of a processive motor as it is able to translocate over distances greater than a single ATP-driven step [8]. This is in contrast to nonprocessive motors, which undergo single translocation events before detaching. To support translocation over large distances, these motors must, therefore, work in populations of several hundred, all within the vicinity of an actin filament to ensure continuous sliding [8]. Using fluorescent labeling, the mean length of a processive run for myosin V has been estimated at 2.4 *μ*m, or, approximately, 66 steps of 36 nm [9, 10].

**Figure 1:**
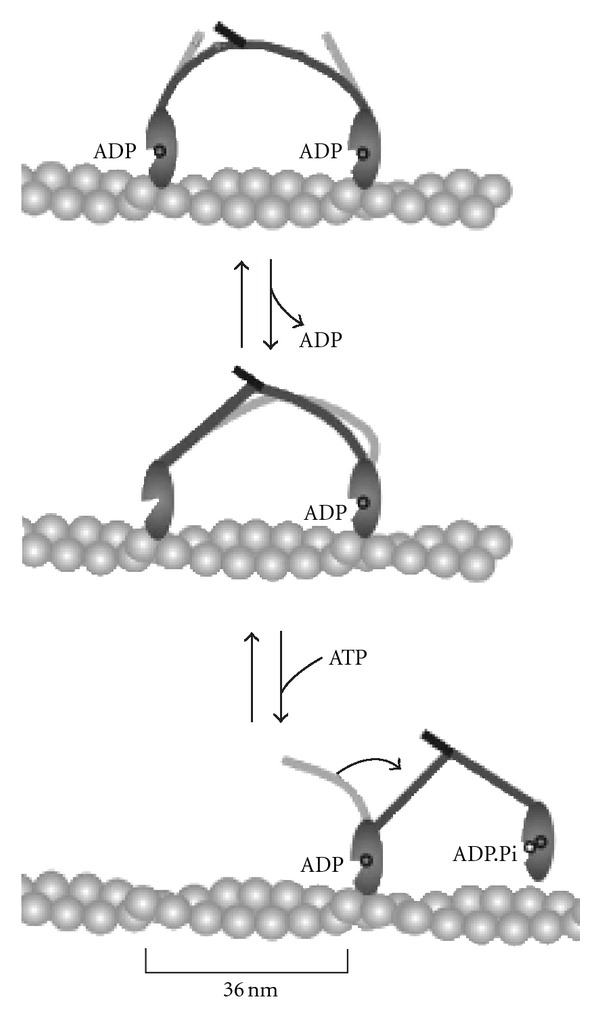
Myosin-V ATP cycle. (a) Both myosins bind ADP and are attached to actin (creating some bending strain from the relaxed “leg” positions shown in light grey). (b) With loss of ADP in the left leg, the power stroke can progress in the right leg. (c) On ATP binding, the left leg is released from actin and swings to the right and with ATP hydrolysis, it to returns to the pre-powerstroke conformation. (Reproduced from [1], with permission).

The mechanical arm movement during myosin walking is coupled to the chemical cycle of the motor and a simplified model for the processive movement of myosin V is shown in Figure 1. Arbitrarily starting with both heads in an ADP bound state, the trailing head, in a postpowerstroke conformation, releases ADP which is the rate limiting step in the cycle. ATP then binds to the trailing head, causing it to dissociate, allowing the leading head in a prepowerstroke conformation to complete its powerstroke. As it does so, the detached trailing head is thrust forward, hydrolyzes its ATP, and reprimes its lever arm to a pre-powerstroke position. This free head makes a diffusive search to bind to the next actin site, becoming the new leading head [11, 12]. Alternate kinetic branches and pathways to this general model have been proposed, differing in the order that the two heads adopt different nucleotide states and their affinity for actin in these states [13–16].

The duty cycle is a concept related to processivity and refers to the fraction of time a myosin head is in contact with the actin filament during a processive run. Given that the affinity of myosin V for actin is strongest during the ADP nucleotide bound state and that ADP release is rate limiting, myosin V spends most of its kinetic cycle bound strongly to actin and is thus classified as a high-duty ratio myosin. It is thought that a high duty ratio (>0.5) is required for all processive myosins to prevent dissociation from the track during movement on actin. Duty ratios can be calculated in a number of ways, such as characterizing the kinetics of the myosin catalytic cycle and using experimentally derived rates or observing actin attachment by single fluorescently labeled myosin. In the case of myosin V, experimental studies on single-headed S1 fragments suggest the myosin is in the strongly bound AM-ADP bound state for approximately 80–90% of the time [17] and a duty ratio over 0.9 for the native myosin dimer [18].

### 1.3. Outiline of the Work

In this work, we use a coarse-grained molecular model of a myosin V dimer with an actin filament [19] to simulate and analyse the dynamics of processive motion.  Section 2 reviews the algorithm used to perform simulations and the type of information generated by the model.

 Extensive additional details on the method, and the current application in particular, can be found in the Supplementary Material. It should be remembered throughout that our modelling approach is not based on molecular physics but employs extended objects including spheres, ellipsoids, and cylinders and should be viewed as a mechanical model. The results generated by the simulations focus on measurable features of the model that can be compared, where possible, to their experimental counterparts. Using this simple model, we attempt to characterise what aspects are important in generating processive motion.

## 2. Methods

### 2.1. Molecular Dynamics Algorithm

The algorithm and model used for our simulations has been described in detail elsewhere [19, 20] and will only be summarised here. Full details of the basic method and the specific data structures used in the current work can be found in the Supplementary Material, available at doi:10.1155/2012/781456. The component that has been developed further in this work is the dynamics of the interaction between actin and myosin which will be described in greater detail.

#### 2.1.1. The Molecular Model

The molecular models were based on the structure of myosin-V (PDB code: 2dfs) [21] using a very course-grained representation that is based on secondary structure elements (SSEs, *α*-helices and *β*-strands) reduced to their axial line segments (sticks). Each end of a SSE stick is represented by a sphere and SSEs are grouped into domains that, in turn are grouped into molecules. Details of the break-down of both actin and myosin (with its light-chains) can be found in [19]. Molecular motion is simulated in a simple Brownian-like manner with each level in the molecular hierarchy experiencing a random displacement and rotation of fixed size with no inertia, experiencing only basic steric exclusion [20].

The structure of the molecules is maintained through pairwise interactions at the SSE and domain level. These include restraints to maintain the internal structure of the myosin head-group and the extended light-chain bound arm (referred to previously as the “foot” and “leg,” by analogy with the myosin V walking motion). There is only a single restraint between the foot and the leg parts and also between the two myosin molecules giving freely rotating ankle and hip joints (within the constraints of steric hinderance).

**Figure 2:**
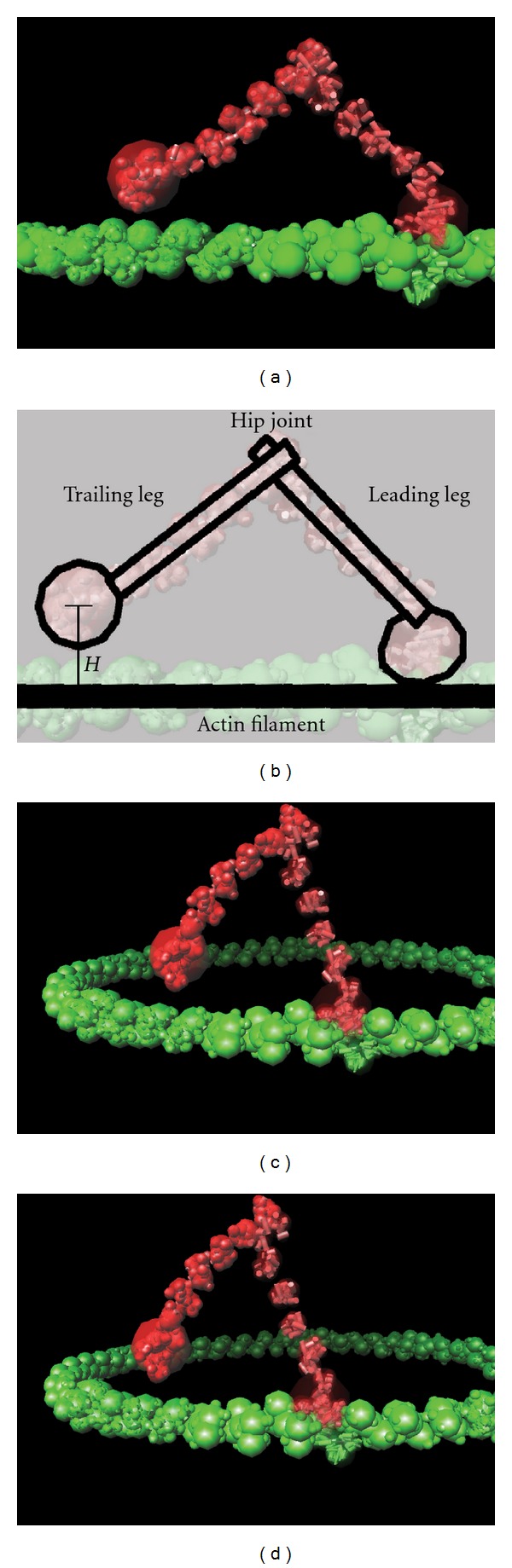
Actin myosin binding animation. (a) The myosin-V dimer model (red) is shown bound to the actin filament model (green). The actin polarity runs from right (− or “pointed”) to left (+ or “barbed”) so the myosin “walks” from left to right. Secondary structure elements (SSEs) are depicted as cylinders (with large/small diameters for *α*/*β*) connected by fine lines. The translucent spheres show the higher level groupings of SSEs into domains. (b) Annotates part (a), with the myosin *leading leg* bound tightly to the actin in prepowerstroke conformation. The myosin *trailing leg* has just detached from the *actin filament* (solid line) and is now free to pivot about the *hip-joint* between the two legs. The height (H) of the free myosin above the filament determines when it is recaptured, initially into a loose-binding mode. Parts (c) and (d) capture the myosin in a similar conformation showing the circuit of 104 actin molecules (52 dimers with 8 half repeats) over which the myosin can move.

The actin component was modelled as a well-restrained filament of 52 actin dimers and to avoid end-effects, these were bent into a circle with the ends joined without any discontinuity in the twist of the filament (Periodic boundary conditions are easily implemented with point objects such as atoms. However, except at the lowest level, our objects are extended ellipsoids and cylinders and, while a PBC could be implemented, this would not be trivial. The actin ring avoids this problem at the cost of minimal local distortion.). This resulted in a ring with a diameter just over 90 nm comprising four full periods of the filament repeat, or eight half repeats with close to 36 nm period taking account of the symmetry of the actin dimer. This ring was large enough to prevent the myosin (with a double-leg span of 40 nm) from stepping across the ring (Figure 2). 

The starting position of the myosin dimer was based on the actin and myosin positions observed in the model of the insect flight muscle (PDB code: 1o1A) [22] with the coordinates of the myosin-V globular (foot) domain superposed on the corresponding domain of the myosin-II in the muscle structure. This places one myosin molecule close enough to be immediately captured and enter a loose-binding mode (described in detail below) with the other remaining free.

Several simulations were run using three myosin V model structures differing only in the number of IQ motifs (light-chains) they contained. These models will be referred to below as myoV-nIQ where n represents the number of IQ motifs present in each heavy chain of the model. From the 6IQ model described previously [23], the coordinates beyond the 2nd and 4th IQ motifs in each chain were discarded to obtain 2 IQ and 4 IQ myosin models, respectively. The myoV-2IQ, myoV-4IQ, and myoV-6IQ were all simulated using the algorithm described above on an identical actin filament model.

#### 2.1.2. Actin-Myosin Binding

The actin-myosin interaction was modeled at two levels, initially by a nondirectional attraction referred to as “loose-binding,” then from loose binding, the interaction could progress to a tighter, directional interaction referred to as “*tight-binding.*” Only in the latter mode was the myosin powerstroke allowed to progress.

The loose binding restraint was activated when the centroid of a myosin head-domain (foot) came within 15 nm of the filament axis. (At this stage, rotational freedom remained undamped.) An initial weak attraction was then activated between the myosin foot and the centre of the closest actin dimer, along with a five-fold reduction in the size of the random displacement experienced by the myosin molecule. These restraints were only applied on a single time step and were reevaluated on every time step, allowing the myosin to alter its choice of actin binding partner.

If the myosin foot approached the filament, additional restraints were added between the two myosin actin-binding domains and the actin dimer centre, introducing polarity to the myosin approach relative to the actin filament but with no preferred orientation with respect to the filament direction or angle of approach. If the orientation of the myosin was favourable, with the two binding domains closer to the actin than the foot centroid, then an attempt was made to move towards tight binding. Otherwise, if the myosin was still close to the actin, it remained in loose-binding mode experiencing now ten-fold reduced random rotation and no random translational motion.

The restraints for tight-binding were applied at the domain level between the myosin-binding domain of the actin molecule that was closest to the myosin foot and its two actin-binding domains. To introduce orientation with respect to the filament axis, the equivalent myosin-binding domains on adjacent actins were used to provide additional restraints. Following the foot analogy, the binding domain at the “toe” of the myosin foot was attracted to the binding domains of the central actin and the actin towards the plus (rough) end of the filament, while the binding domain at the “heel” of the foot was attracted also to the central actin and the actin towards the minus (barbed) end of the filament. From simulations of static binding [19], it was found that the local restraints described above were not sufficiently strong to hold the orientation of the myosin dimer, the other half of which may experiencing large random motions. Increasing the strength of the restraints was not practical as this led to disruption of the actin filament (which experiences a compensating reaction of equal strength). The solution adopted was to introduce a nonphysical displacement that corrected deviations in the myosin foot alignment with the filament axis and its orientation away from the filament. Referring to these motions by the aeronautical terms of “yaw” and “roll,” respectively, the remaining degree of freedom (“tilt”) was left uncorrected, being determined only by the local tight-binding restraints described above.

In tight-binding mode, the random rotational movement of the molecule was further damped by a factor of ten with still no random translational motion. This leaves the myosin molecule almost static with only a slight “wobble” movement and small displacements resulting from the binding restraints and steric repulsion (which is always active).

#### 2.1.3. Powerstroke Motion

The powerstroke motion which involves a rotation of the myosin “leg” by 70° about the “foot” centroid (described in detail previously [19]) was only activated once the myosin was in tight-binding mode and had a reasonable altitude in both yaw and roll (within ±1 radian in each). The powerstroke was not made in one time-step but in steps of 0.05 radians (2.86°), and at every step in the swing, the possibility existed that the myosin would lose alignment or tight-binding and revert to loose-binding from which it might even detach from the actin.

The reverse motion in which the foot readopts the prepowerstroke conformation (associated with ATP hydrolysis) occurred in the model only when the myosin was unbound. As this is not affected by orientation relative to the actin filament, it proceeded at double the powerstroke rate (0.1 rad./step) during which period the myosin was forbidden to bind to actin.

#### 2.1.4. Myosin Dimer Hinge Motion

All the restraints and conformational changes described above apply equally and independently to both halves of the myosin dimer. The only link between the two halves is through the way in which their hinge point is maintained. This hinge is based on a pair of virtual points placed at the midpoint between the two distal domains on each leg in the starting (native) conformation of the dimer. (This is equivalent to a rotation about the hinge for each half combined with a joint translation of both).

When both halves are unbound, after each independent random rotation and translation, the two halves are translated to restore a common virtual hinge point. Such a motion would be completely disruptive of the interaction with actin if either half were bound and in the situation where one half is bound and the other free, the hinge restoration was applied only to the free half.

When both halves are bound, the situation is more complex and if one half is in tight-binding mode and the other in loose-binding mode, then the hinge restoration shift was applied to the loosely bound half only. If both are in loose or both are in tight-binding mode, then a random choice was made. We also coded the option to bias this choice by the length of time each half had been bound, but this was not implemented in the results described below. Independently of the hinge restoration shifts described above, the virtual hinge points were also linked by a restraint to each leg and to each other, providing an additional elastic component to maintain the hinge. If one myosin is free, these restraints will be quickly satisfied but when both are bound, then strain will be accumulated in the myosin legs by the effect of repeatedly enforcing both hinge and binding restraints.

#### 2.1.5. Relationship to the ATPase Cycle

The state of ATP/ADP was not specifically modelled but the conformational changes have clear counterparts to stages in the ATP/ADP binding and hydrolysis cycle. In the free, ATP-bound myosin, hydrolysis of ATP corresponds with the restoration of the myosin conformation to the prepowerstroke conformation and its reacquisition of actin binding affinity. The ADP-Pi bound state pertains throughout the loose-binding mode until the transition to tight-binding. This is marked in the model by the alignment of the myosin and the actin filament within the proscribed tolerances and in biology with the loss of Pi (possibly associated with a similar attainment of accurate binding). The powerstroke follows and the period spent in the postpowerstroke conformation is a tightly bound state in both model and biology, referred to as the “rigor” state. This ends with the release of ADP, which is replaced by ATP resulting in the detachment of myosin from the actin, completing the cycle. We have no explicit link with strain in the molecule to the release of ADP but any strain in the model will disturb the binding conformation making a return through loose-binding to release more likely.

### 2.2. Simulation Setup and Data Collection

The trajectories generated during a simulation recorded information on every computer time-step (or frame number) including the coordinates for both the actin track and myosin dimer in both the reference frames of the actin and the myosin. We also recorded the binding state of each myosin foot as *loose, tight* or *free*, as well as the identity of the actin domain to which it was bound and whether it had attained its postpowerstroke conformation (*swung* state). In addition, since the actin track was circular, the number of laps the myosin has travelled was also recorded.

#### 2.2.1. Staircase Data and Velocity

Distinct steps in the motion of a myosin dimer during a simulation can be identified from a trajectory by monitoring its interactions with the actin filament. If the position of a myosin foot is defined as the site on the actin filament to which it is bound, plotting the position as a function of time (or frames during a simulation) reveals a myosin walk that occurs in distinct step-like increments. To obtain these, given the circular actin track used in the simulations, the position of each myosin head was calculated using the number of laps the myosin had travelled in addition to the current actin of attachment, giving the distance (*D*) as *D* = *C* + (*L* × *N*) − *S*, where *N* is the total number of actins comprising the circular track (52), *L* (Lap Number) is the number of times a myosin head has travelled around the circular track, *S* is the starting actin position, and *C* is the number of the actin bound at the end of the simulation.

#### 2.2.2. Measurement of Step Size

To measure the step size of myosin, frame data from each trajectory was used to extract only those frames where both myosins were attached to the actin track. Given that the duration of a single step lasted over multiple frames, the last frame representing each discrete step was arbitrarily chosen for analysis of step size. The size of the step was then calculated by taking the absolute distance, defined as the number of actin units between the lead and trailing foot for each frame. A frame where one foot was bound on actin 13 and the second on actin 20 would thus result is a step size of 7 units. Taking the average distance between consecutive actin monomers in the 52 monomer circular track gives an average distance between actins of 5.70 nm. This means a step size of 7 units corresponds to approximately 39.9 nm. 

#### 2.2.3. Dwell Times

Dwell times represent time intervals between stepping events, the duration of which in the natural system, are stochastic with an exponential distribution. To extract dwell times in the simulations, we can isolate all steps where both heads are attached to the actin track and count the number of frames where the positions of the myosin feet remain constant. Dwell time distributions were plotted for each myosin head individually and also combined and the resulting histograms were fitted with a single rate constant according to the model described by Yildiz et al. [24], who showed that a plot of the dwell time distribution *P*(*t*) is characterized by an initial rise and then a decline. The derived kinetic equation for this type of movement is *P*(*t*) = *tk* ^2^exp⁡⁡(−*kt*), assuming the stepping rates (*k*) of the two heads are equal. This holds true in experiments where a myosin has only one labeled head and where the label is near the motor domain, such that only the movement of the labeled head is observed. In contrast, if movement is observed for both myosins, the dwell time is reduced to a single exponential:

(1) P(t)=kexp⁡(−kt).

Since the simulations record the position of both heads at a given time, the single exponential equation (1) is the appropriate form. 

#### 2.2.4. Duty Ratios

The duty cycle ratio reflects the fraction of time a myosin head is in contact, strongly bound, with the actin filament during a processive run. To calculate the duty ratios in our simulations, we took the ratio of frames of tight binding to total frames for each head and averaged these values over 5 runs for each of the myoV-2IQ, myoV-4IQ, and myoV-6IQ simulations. This gave the ratio for single heads only. Subsequently, to calculate the duty cycle ratio for the double headed species, we took the average of the ratio of frames where either head of the homodimer was in a strongly bound state to the total frames in each simulation.

## 3. Results

The dynamic model described in Section 2 contains no component that would guarantee any processive movement or even that the myosin motor would remain attached to the actin for any length of time. Both legs of the myosin dimer, when unbound, are equally free to swing in the forward filament direction as well as backwards and each can independently detach from the myosin. It, therefore, came as a welcome observation that the model “walked” around the myosin track with very few reverse steps and a relatively small number of molecules “falling” off.

In the first half of this section, we investigate the origin and requirements of this motion by systematically altering aspects of the model. In the second part, we compare the speed and behaviour of the truncated constructs described in Section 2.1.1.

### 3.1. Characterisation of the Method

As with any track sport, we monitored the overall behaviour of the model by the lap-time of the runner: specifically how many time-steps were required to complete one lap of the 52 actin dimer circuit. Also of considerable importance in the assessment of a molecular athlete is how often it falls off the track. We have not attempted to exhaustively optimise the parameters of the model for speed but we adopted any change that gave a significantly improved lap-time, without introducing physically unreasonable behaviour or leading to an excessive rate of detachment. Although we do not know whether an unloaded native myosin-V has evolved towards maximum speed, it provided a simple evaluation criterion to use in the model development.

#### 3.1.1. Binding Affinities

In the simple Brownian-like model of motion employed in the simulation, all displacements have a fixed size so the strength of pairwise restraints depends on how often they are applied, which in turn depends on the cutoff range within which they become active.

The first restraint to be activated on actin—myosin binding is a centroid—centroid attraction at 15 nm. Our initial test on this was to remove it which was found to result in an improved lap-time with less molecules dropping-off. Given the spread of lap-times, it cannot be claimed that this improvement is significant but the test provided no reason to keep the constraint and it was omitted from the default method. From visual assessment, the removal of the constraint resulted in less distortion of the actin filament (due to the opposite reaction to the myosin attraction) which may explain the better performance (Table 1).

The parameter zone (default 12 nm) that determines when the loose-binding interaction is activated (involving the myosin binding domains) was varied from 15 nm in steps of 3 nm down to 7 nm (which is just 1 nm over the separation at which the actin and myosin molecular envelopes collide). Variation of the zone parameter resulted in some improvement at the slightly higher value of 15 nm but as higher values are more difficult to justify physically, a shift in this direction was not accepted. There was, otherwise, little difference in lap-times except for the shortest value (7 nm) which was over 50% longer than times obtained with the default value (Table 1).

The pairwise interdomain restraints involved in maintaining tight binding are less important than the control of the yaw and roll angles with respect to the filament axis. As described in Section 2, the powerstroke is only activated when both these rotations are within a tolerance of ±60° (set by the parameter: align). To test this limit, values for align of 30° either side of this default range were tested. As would be expected, compared to the default value, the stricter limit resulted in a slightly longer lap-time with the looser constraint being slightly faster but with more molecules detaching. These tests provided no clear indication to alter the default value (Table 1).

#### 3.1.2. Myosin Hinge Effects

An important aspect of the model in the generation of processive motion lies in the link between the myosins, especially when one is undergoing a powerstroke conformational change. Using the best model as described above, the myosin was capable of completing a circuit of the track in under 1000 steps (Table 1). It seemed likely that this efficient processive motion might be a direct result of the large conformational change associated with the powerstroke, causing the trailing leg to be released. To test this possibility, we attempted to nullify the direct effect of the powerstroke by allowing the hinge to dissociate, in effect, dislocating the myosin hip-joint during the powerstroke. Implementing this change led to the unexpected behaviour of most of the myosins detaching from the track (Table 1). The reason for this behaviour is not obvious but we suspect that once the trailing leg is free, the sudden restoration of the hinge disrupts the binding of the attached leg.

#### 3.1.3. Weak Legs

The detrimental effect of altering the hinge meant that little information was obtained on the contribution of leg-strain during the powerstroke to the detachment of the trailing foot. An alternative approach to investigate this is to alter the rigidity of the leg to reduce the build-up of strain. As described previously [19], the domains of the light-chains that constitute the leg are restrained with links between adjacent domains and those adjacent-but-one. In addition, two long links between the first and the last domains were added for extra rigidity. When these two long restraints were removed a marked increase in lap time of 20% was observed, indicating that the rigidity of the legs contributes to efficient motion but it remains difficult to determine whether this is caused by faster detachment of the trailing foot or a more efficient search for the next binding site.

#### 3.1.4. Bound State Coexistence

To discriminate these two options, the binding times and step-sizes were analysed and of particular interest is the coexistence of different binding states on the two myosins. A table was compiled of the number of time steps in which each of four distinct binding states coexisted over 10 single lap simulations of the default myosin model with a leg-brace (good-leg) and a model where this was omitted (weak-leg). The binding states were “free” (F), “loose” (L), “tight” (T), and a substate of tight binding, “swung” (S) in which the myosin had attained its postpowerstroke conformation (Table 2).

By definition, there are no counts when both myosins are free (FF) as this causes a termination of the simulation. As would be expected, there are also no counts when both molecules are in a postpowerstroke conformation (SS) since even with the more flexible leg, this would require too great a distortion. Of the other symmetric states, both double loose binding (LL) and double tight binding (TT) are uncommon, with the former being more abundant in the weak-leg simulations.

**Figure 3:**
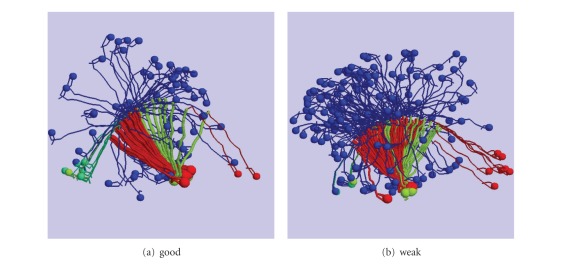
Myosin steps. Superposed frames of myosin molecules retaken from a simulation with (a) a stiff leg (good) and (b) a more flexible leg (weak). In both parts, a line connect the mid points of sequential domains which is drawn thicker in the bound myosin with one of the binding domains marked by a sphere. The colours represent active powerstroke (green), postpowerstroke conformation (red), unbound (blue), and loosely bound (cyan).

As the two legs are treated equally under the model, symmetric counts were added (e.g., ST + TS) and tabulated together (Table 2, *combined*). The differences in these counts (Table 2, *difference*) shows an overall increase because of the longer simulation times for the weak leg, with the greatest increase in combinations where one leg is free and the other bound, and in particular, bound in the postpowerstroke conformation. By contrast, there is no significant change when the two legs are both in tight binding mode (TT), which might be expected if it were easier to maintain this combination with weak legs. Together, these observations suggest that the main difference is not associated with stress between two bound states but with an increased waiting time for the more flexible free leg to find a binding site compared to the stiffer free leg. This can be rationalised in terms of the space searched by each free leg: a stiff leg will search a thin shell, whereas a more flexible leg will search through a thicker shell (Figure 3). As the latter has a greater volume, it will take longer to find the next actin to bind to.

#### 3.1.5. Forced Ratchet Model

The propagation of the powerstroke motion through the myosin hip-joint hinge is the only communication that occurs between the two myosin monomers, which otherwise operate under an identical set of constraints. Except for this physical “force,” there is no instruction that directs the trailing-leg of the dimer to release when the leading-leg becomes bound. To investigate if direct communication between the binding states on the legs might improve the efficiency of the processive motion, we added the condition that when the leading-leg attained binding with the trailing leg in its postpowerstroke configuration, then the trailing leg would be set to the unbound state. This change did not involve any immediate change in position for the trailing-leg but with the other leg in binding mode, then random motion, including the maintenance of the hip joint, would be focused on the trailing leg so giving it a good chance to move away from the actin filament.

An analysis of the coexistence of bound states under this model revealed the same percentage occurrence of both heads in tight-binding mode (TT) as under the default model (Table 2, *good leg* counts) but with a five-fold reduction in states where one of the bound legs was in its postpowerstroke conformation (TS or ST). Although these counts are indicative of a more efficient transfer of motion from one leg to the other, the average lap-time (over 20 circuits) was insignificantly different from the current default model and was, indeed, slightly slower. This unexpected result can be rationalised by the fact that the myosin velocity in our model is dominated by the time taken to search for the next actin binding-site with almost 90% of the time spent in a state with one leg free. Improvement in lap-time can, therefore, only be made in the remaining 10% of the time. As there is already an efficient transfer of motion from one leg to the other under the default model, any additional gains can only be small.

### 3.2. Simulations with Different Leg-Lengths

#### 3.2.1. Staircase Data and Velocity

**Figure 4:**
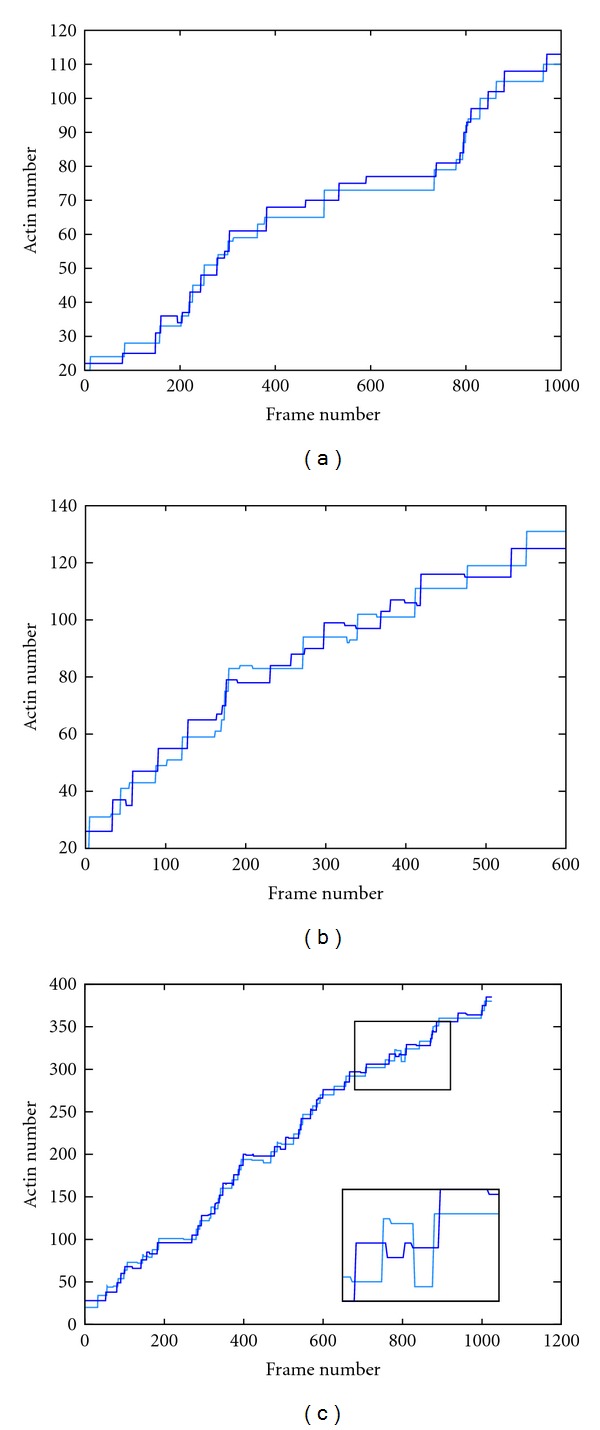
Step traces. These are sample step traces for each of the (a) myoV-2IQ, (b) myoV-4IQ, and (c) myoV-6IQ simulations showing typical staircase events. Trajectories for head 1 in are in dark blue, and head 2 in light blue. A clear example of a backstep is shown enlarged in part (c). *X* axis is frame number, while *Y* axis is actin position/number. Note the difference in scales.

Steps in the motion of a myosin dimer can be distinguished as jumps in the observed amplitude of the staircase-plot of distance against time. The displacement traces for the myoV-6IQ trajectories contain the most frequent staircase events, while those for the myoV-2IQ trajectories contain the least. For all simulations, displacements were biased in one direction, with only occasional backsteps (Figure 4). However, there was a difference in the number of backsteps between the myoV-2IQ, 4IQ, and 6IQ simulations, with the latter two having approximately 4 times as many backsteps. With a total number of steps over all trajectories counted as 1261, 845, and 964 for the myoV-2IQ, 4IQ, and 6IQ simulations, respectively, the backsteps observed accounted for 5.95%, 19.5%, and 20.7% of the total steps. 

**Figure 5:**
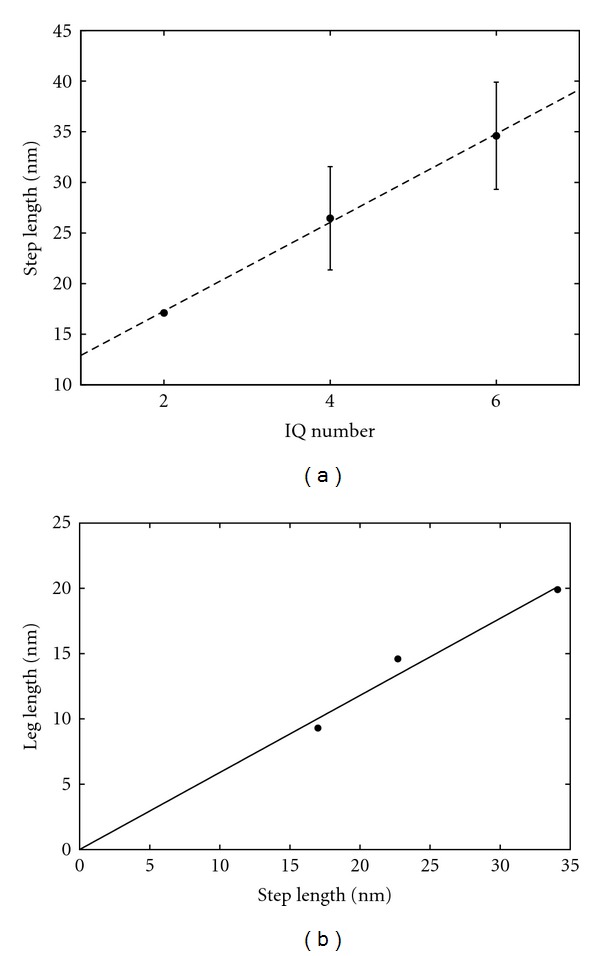
Cumulative step plots. (a) The position on the actin track (*Y*-axis, actin units) for a single myosin leg is plotted at each frame (*X*-axis) for the three models with differing leg lengths: myoV-2IQ (red), myoV-4IQ (green), and myoV-6IQ (blue). The positions include both loose and tight binding modes. (b) All data points over all simulations for the three models (coloured as in (a)) over the initial 3000 frames or 2 laps (104 actins) if shorter, with solid lines in same colors indicating lines of best fit to the data, respectively.

The step traces for a sample of long runs are plotted in Figure 5(a) for each of the three models. It can be seen that the two longer-legged models (Myo-4IQ and Myo-6IQ) are capable of travelling over extended distances, or for the shortest model (Myo-2IQ), travelling less but for a considerable period of time. The points at which the runs terminated give some idea of the length of a processive run which for the 6IQ model is comparable to the 66 steps estimated by Sakamoto et al. [10]. However, we did not use this direct approach to measure processivity since, besides the long simulation times required, after a large number of circuits, the actin track becomes increasingly distorted which can increase the chance of detachment.

To minimize any effects due to damage to the actin track, the velocity of each model was calculated based on a larger number of shorter runs comprising two laps of the track.  Figure 5(b) shows data points collected from all trajectories over the three varying IQ length models with a line of best fit to the data. The fits (in actin-units/frame) are as follows for the myoV-6IQ, myoV-4IQ, and myoV-2IQ models, respectively, 0.057, 0.039, 0.0067. The increase in velocity is directly proportional to arm length only between the 4 and 6 IQ models with the 2IQ model running unexpectedly slowly.

#### 3.2.2. Measurement of Step Size

**Figure 6:**
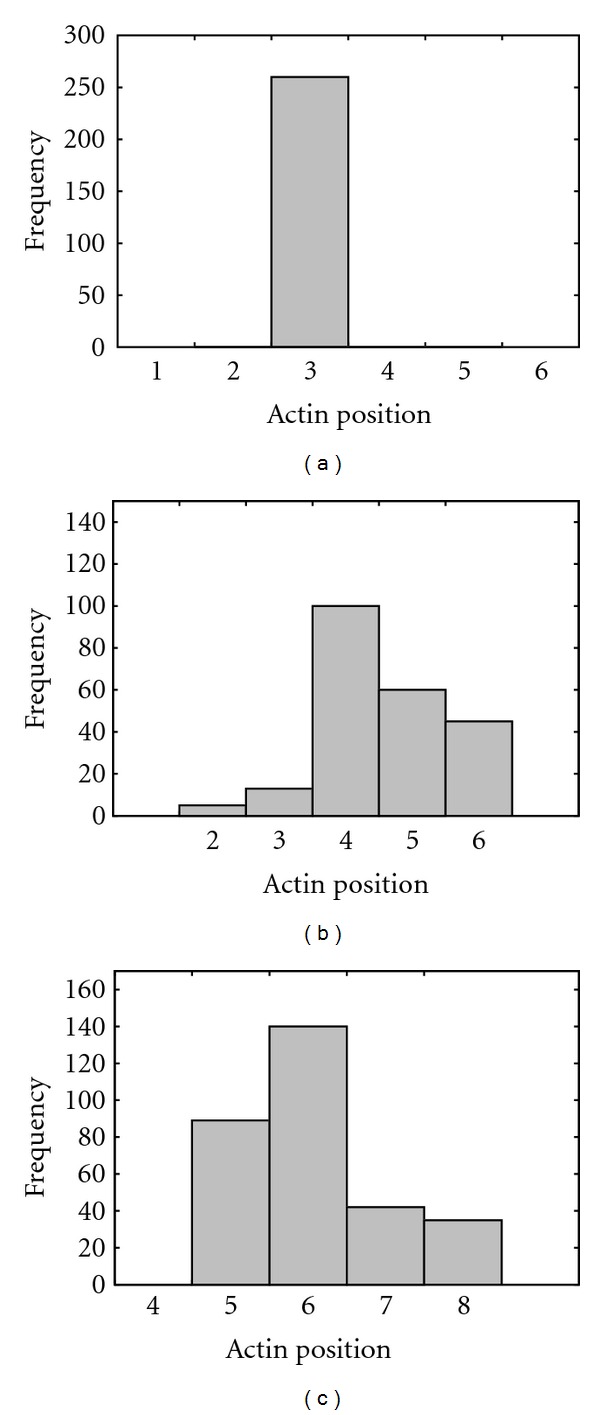
Step Size Histograms for the Movement of MyoV. Plots are for myoV-2IQ (a), myoV-4IQ (b), and myoV-6IQ (c). *Y*-axis shows the number of events (*F*). *X* axis shows the number of actin units separating the two myosin heads of the dimer when both are bound to the actin track. Separation by a single actin unit represents a distance of roughly 5.70 nm. Fit to a Gaussian distribution as follows: mean ± s.d., 2IQ = 3.0 ± 0.0, 4IQ = 4.64 ± 0.93, 6IQ = 6.07 ± 0.95.

**Figure 7:**
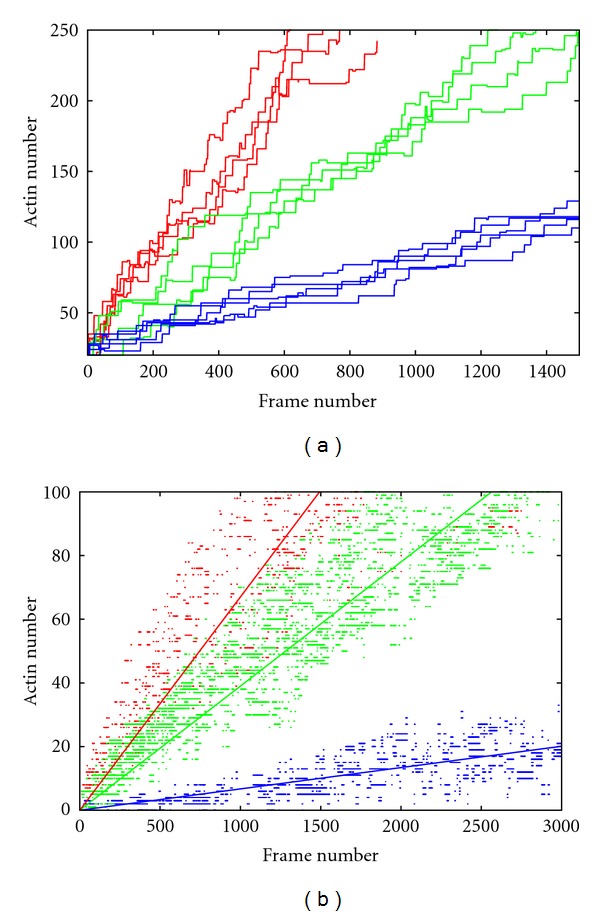
Step-size with number leg-length. (a) Step size (*Y*-axis, nm) against IQ number (*X*-axis). Note that there are no error bars for 2IQ as every step size was identical at 3 actin units of separation. (b) Step size (*Y*-axis, nm) against Leg-length (*X*-axis, nm). The models had legs ranging between 9.3 nm (2IQ) to 14.60 nm (4IQ) to 19.90 nm (6IQ) in length.

The average step sizes for the 2IQ, 4IQ, and 6IQ myosin simulations obtained from the fitted Gaussian distributions were 3.0 ± 0.0, 4.64 ± 0.93, 6.07 ± 0.95 units, respectively (Figure 6). In contrast, using median values from the data gives average step sizes of 3, 4, and 6 units respectively. Given the relation of 5.70 nm per 1 actin unit, these values translate to average step sizes of 17.1 ± 0.0 nm, 26.45 ± 5.30 nm, and 34.60 ± 5.42 nm using the means, and 17.1 nm, 22.8 nm, and 34.2 nm using the median values. A plot of the step sizes versus the number (*n*) of IQ motifs (Figure 7(a)) shows a linear relationship with a line of best fit of 4.38*n* + 8.52 between the step size of the myosin models (myoV*n*IQ).

Plotting the step size against actual leg-length (*L*) also reveals a close to linear relationship with a best fit line of 0.598*L*-0.164, which almost passes through the origin. When constrained to include the origin, the slope changes slightly to 0.59 (Figure 7(b)). This implies that there is a constant angle formed between the myosin legs when they bridge their binding sites, irrespective of leg-length. If the leg-length is *L* with stepsize *s*, then this angle (*θ*) is 180 − 2cos⁡^−1^⁡(*s*/2*L*), which for *L*/*s* = 0.6, gives *θ* = 112.9°, or 33.55° between the actin filament axis and the myosin leg. If we assume a movement of 70° between the pre- and postpowerstroke conformations, this positions the bound myosin almost orthogonal to the filament axis before the powerstroke. 

#### 3.2.3. Dwell Times and Duty Cycle Ratios

**Figure 8:**
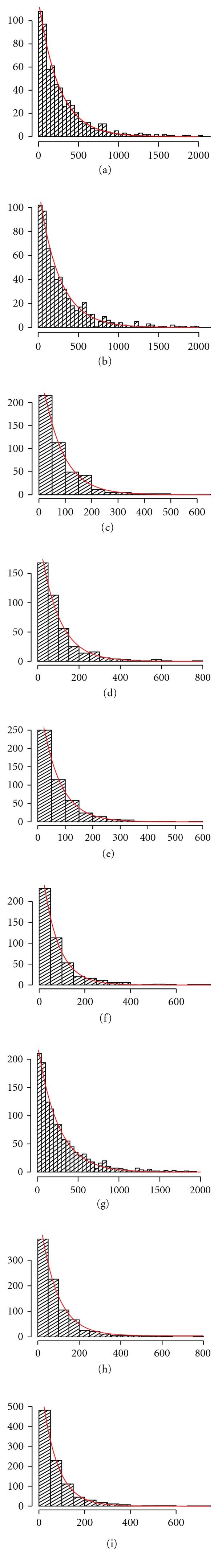
Dwell Times Distributions. *X*-axis shows number of frames, *Y* axis shows number of occurrences. Plots (a, c, e) show distributions for first head of myoV-2IQ, myoV-4IQ and myoV-6IQ simulations and (b, d, f) for the second head of the simulations, respectively. Plots (g, h, i) show the combined data for both heads. All histogram data is divided in equal bin widths of 50 frames. Red lines are fits of (1) to the data. See Table 3 for rates obtained from best fits.

Since the simulation algorithm records the position of both heads at any given time, the single exponential in (1) was used to characterize the distribution of dwell times during a myosin model walk for each of the three different IQ lengths. The theoretical fits to the distributions (Figure 8) gave rate constants summarized in Table 3, where *k*_1_ denotes the rate for head 1, *k*_2_ denotes the rate for head 2, and *k*_12_ denotes the rate from the combined data of both heads. The rates obtained for the fitted lifetimes for each head individually and then combined showed no statistical difference, confirming that both heads have an equal stepping rate, as expected. As such, subsequent analysis was performed using the rates from the combined data from both the myosin heads (*k*_12_) for improved statistics. Using the above rates and theoretical curves gave average dwell lifetimes of 252.7, 83.06, and 67.61 frames for myoV-2IQ, myoV-4IQ and myoV-6IQ, respectively.

The calculated duty cycle ratios for the 2IQ, 4IQ, and 6IQ simulations were 0.51 ± 0.02, 0.55 ± 0.03, and 0.54 ± 0.02 for the single heads, and 0.993 ± 0.001, 0.943 ± 0.001, 0.933 ± 0.002 for the double-headed species, respectively. As will be discussed below in more detail, both the dwell times and duty cycle ratios correspond well with experimental data.

## 4. Discussion

### 4.1. Behaviour of the Model

The mechanical model employed in the current work retains a close correspondence to the known structure of myosin-V, with the exception of the junction between the two myosin molecules (the “hip” joint) which is unresolved [21]. The degrees of freedom within this model have been previously described and parameterised to recapitulate the internal molecular motions and the relative orientation of myosin to the actin filament when bound [19]. The dynamic behaviour developed in the current work resulted from the additional capacity for both myosins to bind to actin and progress through a powerstroke on binding. As a simple consequence of having relatively stiff legs, both myosins cannot remain bound when they are both in their postpowerstroke conformation. This means that when one leg is bound in the postpowerstroke conformation and the other binds and attempts a powerstroke, one or the other must detach. Our model contains no explicit action or bias to specify which myosin that should be and there is a 50 : 50 chance it could be either the leading-leg or the trailing-leg. That this apparently symmetric situation should lead to largely unidirectional processive motion along the filament can be explained by the differing pre- and postpowerstroke states of the two myosins. If, by chance, the leading leg detaches, then because the trailing leg has already undergone a powerstroke, this means that the leading leg will remain in the proximity of the filament and is able to rebind. However, if the trailing-leg detaches, then the leading leg is free to continue its powerstroke which will swing the trailing leg away from the filament, reducing its chance to rebind.

The processive motion that results from this asymmetry is relatively insensitive to the parameters of our model. When behaviour is monitored by lap-time and falling-off, the choices that make most difference are those that affect the search time for the unbound leg to find a new actin binding-site. These include how close the myosin foot must be to an actin before it is captured in loose-binding mode and how stiff the leg is. The latter dependency appears to be a consequence of the greater efficiency of searching in a wide arc rather than spending time in the vicinity of the current binding point. Setting aside considerations of efficiency, the essential requirement in our model to generate processive motion is that it should be physically impossible (or very unfavorable) for both myosin monomers to exist in a postpowerstroke conformation and both be bound to actin. Without this condition, both myosin legs would bind, swing into postpowerstroke conformation and stick forever. In addition, it is also necessary for each foot to occasionally slip from tight-binding since, obviously, if tight-binding were permanent, no motion could result.

Any mechanism to allow the incorporation of very tight binding into the model would require communication between the legs on the binding state of their feet (or heads). In this ratchet-like model, the trailing-leg must release from the actin (or loosen its grip) when placed under strain by the powerstroke swing of the leading-leg. Such a mechanism may indeed exist in the natural system as it can be postulated that conformational changes induced by strain might lead to a weakening in the affinity of the trailing leg to bind ADP. To test this model, we directly encoded communication between the myosin molecules so that a trailing leg in postpowerstroke conformation would immediately release when the leading leg bound. This did not lead to any increase in the myosin velocity or affect the fall-off incidence and would suggest that a ratchet mechanism is not necessary for processive motion. However, our model differs from nature in having the search for the next binding site as the rate-limiting step, rather than the release of ADP from the bound myosin. It may be that if placed under load, stronger binding would be needed in our model to prevent the detachment rate from increasing, which in turn might introduce the need for a ratchet mechanism.

### 4.2. Leg-Length Variation

#### 4.2.1. Step Size

To examine the role of the leg length in determining step-size, IQ motifs were subtracted to create “mutant” myosin V models with shorter neck regions. The step size distributions of the 2IQ-, 4IQ-, and 6IQ-HMM showed a linear correlation (Figure 5) between the step size and neck length, which ranged from *x* nm (2 IQ) and *y* nm (6 IQ). This suggests our model is consistent with the experimental studies that showed the working stroke of myosin V is a function of the length of its leg (or lever arm) and that step size during a processive run is dictated primarily by the length of the leg (or neck) and not solely by the pseudorepeat of the actin filament [4, 6, 25, 26].

#### 4.2.2. Processivity

In all the simulations for each of the 2, 4, and 6-IQ myosin models, the myosin seldom fell off the track. This meant that we could not calculate processivity from the number of steps to dissociation. Instead, the maximum distance travelled during the simulations provided a lower limit as to the processivity of the myosin model. The 2IQ, 4IQ, and 6IQ myosin models were able to travel across 200, 250, and 500 actins, corresponding to 1140 nm, 1425 nm, and 2850 nm, respectively, before termination. These distances are in the micrometer range, corresponding to the 2.4 *μ*m mean processive length reported in the literature for myosin V, or approximately sixty six 36 nm steps [10]. The differences in maximum distance travelled prior to termination may appear to correlate with leg length, but this corresponds only to an arbitrarily different number of runs around the track. It is likely that the 2IQ-myoV model could also achieve a similar distance to the 6IQ model.

### 4.3. Staircase Data and Velocity

The longer-legged models (4 and 6 IQ motifs) moved with an average velocity that was proportional to their leg-length whereas the average number of time frames taken by the 2 IQ length simulations was proportionally greater (Figure 5 (b)). A linear relationship between leg length and velocity is not necessarily expected since velocity depends on the amount of time needed to search for the next binding site. However, this does not offer any immediate explanation for the slowness of the 2-IQ model and examination of the molecular motion suggested that for this model there was a disproportionate amount of time spent searching in the direction of movement. This indicates that although the leading foot (or head) is in the correct region, it has more difficulty forming an attachment. We suspect that this is due to steric hindrance of the adjacent leg, perhaps caused by an over simplified model for the hip joint that becomes exaggerated with the short leg-length.

#### 4.3.1. Dwell Times

The different myoV-nIQ simulations produced dwell times that appeared correlated to the length of the lever arm, with myoV-2IQ having the largest dwell time and myoV-6IQ the smallest. The single study that has looked at the dwell times for different myosin V IQ mutants by Sakamoto and colleagues found that their dwell times did not correlate with neck length in any predictable manner [26]. They attribute this result to possibly distortion in the shorter IQ mutants (2IQ and 4IQ) caused by intramolecular strain as the heads bind at different azimuthal angles, and loss of such strain in a longer IQ mutant, thus altering the strain-dependant nucleotide release rates from the wildtype 6IQ myosin V in their study [26]. As there is no explicit link between strain and nucleotide release in the course grained myosin V model, one possible explanation for the correlation of dwell time to arm length in the simulations is that the longer the arm, the quicker the potential for distortion and strain buildup in the arm that can in turn disturb the binding conformation of the myosin to the actin filament making its release more likely.

In addition to a relative comparison between dwell times for varying IQ lengths, it is possible to use both the rates obtained and experimentally derived rates to obtain a rough estimate of the timescale in our simulations. It should be noted, however, that dwell times are not an absolute property of the myosin V motor, but rather vary depending on the environmental conditions of the system under study. Differences in whether or not the myosin V under study is monomeric or dimeric, the amount of external load/forces applied, if any, and (assay) reagent concentrations all result in the array of dwell times reported in the literature. To compare dwell time rates from the coarse grained simulations to experimentally derived kinetic rates, it is, therefore, important to select those from studies/experiments employing a kinetic model that best resembles our coarse-grained model.

The two crucial parameters in the simplest kinetic model described in the literature, which coincidentally best matches our coarse grained actomyosin model, are the ATP and ADP conditions, as the relative concentrations available of each nucleotide will alter the kinetic reaction rates of processive motion. In saturating ATP conditions (the implicit conditions in our model), ADP release has been shown to be the rate limiting step in the ATPase cycle, and the motor predominantly dwells in a state waiting for ADP to dissociate. The rear head then releases nucleotide and binds an ATP causing it to dissociate from actin. In studies/kinetic models where ADP release is rate limiting, the reaction rate constant has been consistently calculated at 12–14 sec^−1^. With the ADP release rate being directly coupled to dwell time (a slower rate causes longer dwell durations), it can be conveniently used to convert the virtual time to an estimate of real time. By calibrating relative to the rate limiting step using the myoV-6IQ simulation, a single frame or step would correspond to roughly 1.2 seconds.

#### 4.3.2. Duty Cycle Ratios

Biologically, a high duty ratio is critical for processive movement because it ensures that at least one of the two heads of a myosin V molecule is strongly bound to the actin filament at any given time, thus ensuring that random thermal forces do not cause it to diffuse away from the track [27]. For processive motors like myosin V, the duty ratio must, therefore, be greater than 0.5 at a minimum. Studies of single-headed recombinant myosin V have shown that its ATPase cycle has a duty ratio of around 0.7 [18, 28], with some suggesting duty cycle ratios reaching up to 0.9 [17]. These values are explained by the underlying kinetics, where ADP release as the rate limiting step (under saturating ATP conditions) causes the myosin V head to spend the majority of its steady state cycle strongly bound to actin, and thus the higher duty cycle. This is in contrast to myosin II, whose kinetics are distinct from that of myosin V. Specifically, the rate limiting step in the ATPase cycle of myosin II is either ATP hydrolysis or Pi release and so the predominant state is weakly bound or detached from actin.

The duty ratio can be estimated from the ATPase cycle rate constants determined in solution, if the cycle is fully characterized by solving the steady-state distributions of all the actin bound states. Comparing the rate constant for ADP dissociation from the A.M.ADP state to the overall *k*_cat_ for the steady-state ATPase gives a measure of the proportion of motors in a strongly bound state and hence the approximate duty ratio [8]. Note that this method is not generally applicable across all other classes of myosins where the affinity for actin may differ during an ATPase cycle. Forgacs and coworkers determine the duty ratio by the flux into and out of the strongly bound intermediates (*k*_in_/(*k*_out_ + *k*_in_)) which is limited by the rates of hydrolysis, phosphate dissociation, ADP dissociation and M-ATP dissociate ion from actin [17].

Given a duty cycle ratio measured for a single-headed myosin, then the ratio for the double headed species can be calculated as 1 − (1−*r*)^2^ assuming no interaction of the heads. In our simulations, the 2IQ, 4IQ, and 6IQ heads spent an average of 51%, 55%, and 54%, respectively of their time strongly bound to actin. Using the above formula, this would predict duty ratios of 0.76, 0.80, and 0.79 for the myosin homodimer. However, owing to the fact that each frame recorded weak, strong or off bound state for each head, the duty cycle ratio for our double-headed myosin could be calculated directly by summing the frames over which either head was in a strongly bound state and comparing to the total frame number in each simulation. Calculated in this way, our myosin V 2IQ, 4IQ, and 6IQ models gave duty cycle ratios of 0.99, 0.94, and 0.93, respectively.

## 5. Conclusions

We developed a very coarse-grained model for myosin-V motion along actin that retains sufficient detail to allow direct comparison with experimental results, including the reproduction of different velocities and step sizes with different leg-lengths. Our results confirmed that a high duty cycle is a prerequisite for processivity but whether this obviates the need for a gated mechanism, as suggested by some studies [29], was not fully resolved by our experiments. Our attempt to isolate strain in the myosin dimer hip-joint by allowing dislocation proved to be disruptive and softening the strain by making the legs more flexible, also affected the search for the next binding site. However, the observation that the frequency of occurrence of double-bound states was the same with both stiff and flexible legs suggests that leg-strain was not an important component in the generation of processive behaviour. This conclusion was supported by explicitly introducing direct communication between the feet (replicating a ratchet model) where we found that the small increase in the efficiency that resulted did not lead to any significant increase in speed.

From a methodological point of view, we have developed a novel algorithm capable of simulating Brownian dynamics for large macromolecular systems, amenable to coordinating across several different levels of resolution simultaneously. We chose to apply our method to investigate how myosin V might achieve its processive motion along actin filaments and our model represents the minimum mechanical requirements necessary to do so. While there have been a number of mechochemical models published previously in the literature on the actomyo system, these incorporate both kinetic and structural parameters using chemical transition rates reported in experimental studies and structural details from crystallographic studies to describe the conversion of free energy into mechanical work. In contrast, our hierarchic, coarse-grained model uses only structurally based parameters to describe the actomyosin complex, both as a static and dynamic system. These parameters are sufficient to reproduce the degrees of freedom associated with the flexible connections of myosin and the characteristic processive motion along the actin filament.

## Supplementary Material

The supplementary material describes the implementation of the dynamics method and the specification of the molecular structures analysed in this work.

## Figures and Tables

**Table 1:** Parameter optimisation of the default model. For each change in the model, the mean *lap-time* was recorded (with its standard-deviation) along with the number of falls (when the myosin detached from the actin track). These partial circuits were not included in the number of *runs* used to calculate the mean lap-time. Experiments with the myosin hip-hinge were generally disruptive (see Section 3.1.2). Changing the capture *zone* for loose-binding and the range within which to *align* the actin/myosin orientation produced no significant improvement. The only change that was adopted as the new default was to remove an *initial attraction* between the actin and myosin.

Falls	Lap-time (±)	Runs	Description
6	1019.7 (323.4)	20	Initial model

21	1273.7 (606.1)	6	Hinge fix off in swing
20	1113.3 (753.9)	10	Hinge fix off in swing but strong link
1	1141.7 (510.4)	20	Hinge fix off when equally bound

**2**	**963.4** (266.0)	20	Initial attraction off (new default)

0	974.9 (290.5)	20	Zone = 1.5
0	1086.9 (311.4)	20	Zone = 1.2
1	1051.5 (525.6)	20	Zone = 0.9
0	1566.6 (500.7)	20	Zone = 0.7
0	1108.1 (498.2)	20	Align = 0.5 rad.
3	961.35 (288.1)	20	Align = 1.5 rad.
1	1225.3 (422.1)	20	Flexible leg (weak)
2	1051.8 (383.6)	20	Release trailing-leg (ratchet motion)

**Table 2:** Binding state coexistence in the two myosin “legs.” The binding state of each myosin leg can be either *free*, in *loose* or *tight* binding or in the latter state after the powerstroke has *swung*. Combinations of these four states were counted for both *leg-1* and *leg-2* over ten circuits of the track. Counts were tabulated for the default model (*good leg*) and a more flexible myosin (*weak leg*). As no significant difference was seen between symmetric counts (leg-1/2 and leg-2/1), these were *combined* and the (weak-good) *difference* taken.

Leg-1 state	Leg-2 state			
	Free	Loose	Tight	Swung
	*Good leg*			

Free	0	454	2799	644
Loose	712	109	309	95
Tight	2150	258	103	33
Swung	759	154	49	0

	*Weak leg*			

Free	0	721	2262	977
loose	801	199	435	178
Tight	3190	247	106	39
Swung	1505	163	57	0

	*Combined *(**weak**∖**good**)			

Free		1166	4949	1403
Loose	1522		567	249
Tight	5452	682		82
Swung	2482	341	96	

	*Difference (weak-good)*			

Free	0			
Loose	356	90		
Tight	503	115	−3	
Swung	1079	92	14	0

**Table 3:** Dwell time rate constants. The mean rate constants (frames^−1^), with standard deviations below, are tabulated from fits of the single exponential equation (1) to dwell time distribution histograms (Figure 8) for the first head (*k*_1_), the second head (*k*_2_), and both heads (*k*_12_) of the myosin dimer model for each leg length (IQ units) of the myoV-2IQ, myoV-4IQ, and myoV-6IQ simulations.

Rate const.	*k*_1_	*k*_2_	*k*_12_
2-IQ leg			
Mean	3.909 × 10^−3^	4.004 × 10^−3^	3.957 × 10^−3^
Std.dev.	1.096 × 10^−4^	1.089 × 10^−5^	6.451 × 10^−5^
4-IQ leg			
Mean	1.321 × 10^−2^	1.089 × 10^−2^	1.204 × 10^−2^
Std.dev.	5.476 × 10^−4^	5.228 × 10^−4^	3.192 × 10^−4^
6-IQ leg			
Mean	1.507 × 10^−2^	1.450 × 10^−2^	1.479 × 10^−2^
Std.dev.	1.947 × 10^−4^	3.343 × 10^−4^	2.112 × 10^−4^
