# Supplementary material for: Coarse-Grained Simulation of Myosin-V Movement
Source: Comput Math Methods Med. 2012 May 23;2012:781456. doi: 10.1155/2012/781456 (PMC3366208; doi:10.1155/2012/781456)
Supplement: Supplementary file 1 — The supplementary material describes the implementation of the dynamics method and the specification of the molecular structures analysed in this work. [file 781456.f1.pdf]

SUPPLEMENTARY MATERIAL for a Coarse-grained simulation of myosin-V movement

William R. Taylor*, and Zoe Katsimitsoulia

Division of Mathematical Biology,
MRC National Institute for Medical Research,
The Ridgeway, Mill Hill, London NW7 1AA, U.K.

February 27, 2012

1 Coarse-grained Modelling Method

1.1 Overview

The nature of the macromolecular structure data is hierarchic so it is natural use a hierarchic data structure to capture it. With a view to generality, each unit in the hierarchy at any level should be identical with the distinction between levels being made only through externally specified (user) parameters. The most fundamental aspects to be specified include the shape of the unit, its linkage to other units (such as whether it is in a chain or multiply bonded) and how units interact with each other. For ease of description, a unit contained in a higher level unit will be called its "child" with the higher level referred to as the "parent" of the lower level unit. Units with the same parent are therefore "siblings" and if they form a chain, are designated "sister" (preceeding) and "brother" (following). Anthropomorphic terms based on family relationships will be used through.

Interactions can be distinguished as inter- and intra-level. Within a level (intra) these will either be repulsive (active for a pair of units in collision) or attractive (active between a bonded pair). However, there are no bonds between levels (unless individually specified by the user) and no bumps between levels. Inter-level interaction consists only of coordination of motion and containment. Coordinate movement means that when a parent moves, all its children move too, which by implication, continues down through all successive generations. In the opposite direction, the centroid of the children determines the position of the parent. This relationship also implies indirect interaction between the levels so that when children in different families collide then their parents will also experience a lesser repulsion. Containment was implemented as a simple kick-back to any children that had 'strayed' beyond the shape boundary of their parent.

The motion of each unit is purely random with a fixed step-size defined in

the user-specified parameter file. Every move is accepted whether it violates bond geometry of leads to collisions [Katsimitsoulia & Taylor, 2010]. Clearly this would lead to a degradation in the molecular structure so independently of this imposed motion, the bond lengths and local chain geometry (if there is a chain) are continually refined towards their initial configuration. Similarly, units in collision are also corrected. All these processes run concurrently (implemented as separate threads) and in addition a user specified process also runs in parallel in which the directed elements of the model are specified. All of these processes operate on a single representation of the coordinates so care has been taken to ensure that undefined states do not arise in one process that would disrupt another. In general, this can be avoided with each process working on a temporary copy of the coordinates it needs then writing these in one step back into the structure.

The overall structure of the implementation is shown in Figure 1 along with the names of the processes that will be referred to below. The two processes that maintain the integrity of the molecular structure are the collision detection and correction process and the process that maintains the specified links in the chain: respectively, called the `bumper` and the `linker` which will be considered first.

1.2 Steric exclusion

It is intended that the implementation should be applied to very large systems so any collision detection based on a full pairwise algorithm would be impractical. This is commonly avoided in molecular (and other) dynamics programs through the use of a neighbour list in which each atom maintains a list of its current neighbours and checks only these for collision. This has the unavoidable problem that the list must be revised periodically. We adopted a similar approach except that we used the hierarchic structure of the data to provide built-in neighbour lists where any unit only checks its siblings for collisions. As

each family is usually in the order of 10–100 units, this would not be a large task to compute in a pairwise manner, however, we use a faster approach based on ranked lists of units that are maintained for each dimension (X,Y,Z). As sorting avoids quadratic operations in the number of objects, this is much faster for large families [Taylor & Katsimitsoulia, 2010].

When two units are in collision, they are repelled only if they have no children. Again a single (user specified) kick-back step is applied equally to each unit along the line connecting their centres. If the units have children, then collisions are found between their joint families within a restricted range along the line connecting the two parent centres. If there are no grandchildren, repulsion is again implemented along the line connecting their two centres, otherwise the process is repeated at each lower level until the lowest atomic [1] level is reached.

As mentioned above, parents of different families will automatically adjust their position indirectly to the repulsion between their families as the centroid of the children will move slightly apart. This is often a small effect, and before it becomes significant, the children can become bunched at the collision interface which has the reverse effect of bringing the parents (and hence their children) even closer together. To avoid this, on the return path from the traversal of the family hierarchy (ie: revisiting the parents of the colliding children), the parents themselves are given a small direct displacement proportional to the number of their children that were in collision. If only the positions of the atomic level units were observed, this would have the appearance of a repulsive 'field' as there would seem to be "action at a distance" across a family. Alternatively it can be imagined that the children are embedded in a soft parental jelly-like matrix. Computationally the approach means that in any collision at a high level, there will be fewer low-level collisions generated which saves on computational expense.

This approach to the treatment of collisions has an additional effect in that

[1] The term "atomic" only means the lowest level in the hierarchy, which in the protein applications discussed, is the residue level (based on the α-carbon position).

it is relatively insensitive to the shape of the colliding objects, which in our implementation can be spheres, ellipsoids or tubes (discussed in mode detail below). If we assume that the atomic level consists of spheres, then different shapes at higher levels are primarily defined by the distribution of their children within them which in turn is determined by the shape within which the family is confined. The only discrepancy comes through the point at which units at the parental level detect collision as, for computational simplicity, this is kept as an isotropic test at the radius of their (user defined) bumping sphere. Objects that are long/thin tubes or extremely prolate ellipsoids which are fully contained in a bumping sphere can therefore be considered in collision before any of their family members come in contact. At worst, however, this is just a slight waste of computer time as the count of colliding children will be zero and the parents will not respond.

1.3 Polymers and Cross-linking

1.3.1 Specifying chain connectivity

Unless liquids are bring modelled, the links between units (which are not distinguished from bonds) are the components that impart greatest structure. For biological polymers, links between just adjacent units along a chain, combined with steric exclusion, are sufficient to define a basic model. However, even this, apparently simple, imposition of structure leads to complications in a hierarchic model. If the atomic level is a linear chain, then so too are all higher levels but this is not so if each atomic family forms a separate or a circular chain. Then higher levels can be unlinked (eg; a liquid of cyclic peptides) or otherwise linked in their own way. The linkage polymer state of each level can be specified by the user independently but chain interdependencies are checked internally and imposed by the program.

The structural hierarchy (family structure) is not determined by chain connec-

tivity but should be based on groups of units that will tend to move together as they are all acted upon by their parent's transform operations. At the lower level, such groupings will typically consist of consecutive units along a chain (such as an α-helix), however, at the domain level and secondary structure level for RNA, families of units will also be composed of sequentially discontinuous segments. Computationally, this requires some book-keeping to keep note of which members are linked across families. To facilitate this, each unit holds a record of its preceding unit, referred to as its "sister", and its following unit, referred to as its "brother"; both of which may specify any unit in any family at the same level. Together, the sisters and brothers constitute two linked-lists, with brothers running from the start of the chain to the end and sister in the opposite direction.

As chain connectivity is not restricted by family groupings, its path at the next higher level is not necessarily linear and can be branched. This means that each unit may have more than one brother or sister which is equivalent to branches in the chain in both directions. In general, this specifies a network and to deal with the associated "book-keeping", each unit holds a stack of its brothers and sisters. Following a chain is therefore not simple and when listing a chain in "sequential order", the lists of brothers and sisters are followed recursively from any given starting unit.

1.3.2 Inter-chain cross-links

Polymer chain links are such a common feature of biological macromolecules that the capacity to encode them was included as a general feature in the data structure of each unit. Inter-chain cross-links, which are less ubiquitous, were allocated only as requested by the user in the data file that specifies the model. For any level, a fixed number of links could be specified, not all of which need necessarily be used. If no linking capacity was specified then computer memory was not allocated.

Although links can be individually specified, some automated features were incorporated to ease the burden of assigning the local cross-links associated with secondary structure, both in proteins and RNA. For proteins, two types of secondary structure can be defined: the α-helix and β-sheet. The former is purely local and two links were automatically set to the relative chain positions +4 and -3 of the ideal length found in proteins. Similarly, two local links were made along a β-strand to the +2 and -2 positions. However, each strand in a sheet makes non-local links which can be specified by data provided in the coordinate input specification which is automatically generated by a separate program that calculates the definition of secondary structures.

1.4 Geometric regularisation

Steric exclusion combined with the range of linkage described above can generate a relatively stable structure. However, given a background of "thermal" noise, any less constrained parts of the structure will be free to diverge from their starting configuration under the given distance constraints. Typically, this involves twisting and shearing that can generate large motions with little violation of the specified distances, which in principle, cannot constrain chirality. A general mechanism, based only on local angles and distances was provided to reduce these distortions and was applied equally to all levels that form a chain.

In a chain segment of five units (designated: b2,b1,c0,a1,a2), six distances were recorded from the starting configuration in the upper half of the matrix of pairwise distances excluding adjacent units. Three angles were also recored as b1-c0-a1 and the torsion angles around b1-c0 and c0-a1. These local distances were continually refined as were the angles. Distances can be regularised with little disruption, however, refining torsion angles can sometimes lead to an error propagation with dramatic effects. To limit the potential for this the torsion angles were dialled-up exactly to generate new positions for b2 (b2') and a2

(a2'). These were used to form a basis-set of unit length vectors along: $x = a2' - b2', y = c0 - (a2' + b2')/2$, with z mutually orthogonal. Starting from the centroid of the five points, the coefficients of an equivalent basis-set defined on the original positions were applied to the new basis-set to generate the new coordinate positions. The result is a compromise between angle and position that remains stable over repeated application.

Although only local information is used, its application over all levels leads to a global effect and indeed is sufficient by itself to recapitulate a large structure. As the procedure was designed to correct defects caused by the addition of random motion (caused by `mover` in Fig.1), it was not implemented as an independent parallel process but included in `mover` and applied after the coordinates had been displaced, so keeping a balance between disruption and correction. A final feature was included to allow for the necessary requirement that in a dynamic model, the starting configuration of the structure should not be exclusively maintained. This was accommodated by periodically shifting the target distances and angles towards those found in the current configuration. The overall effect of this procedure is to provide a buffering effect against random motion and is similar to giving rigidity to the structure but still allowing movement under a persistent "force". In the current implementation, this shift is by 1% once in roughly every 100 activations of `mover`. This can be adjusted depending on the application.

1.5 Shape specification

Three basic shapes were implemented: cylinder, ellipsoid and sphere. Although the sphere is a special instance of an ellipsoid, there are implementation details, described below, that make them distinct. Each shape type by itself has elements of symmetry that can make their orientation arbitrary, however, this symmetry is broken when a unit contains children in an irregular configuration. Thus each unit

needs to have an associated reference frame that determines its orientation and is acted on by rotational operations. For a unit in a chain, the current reference frame is based on the direction from its sister to brother (X) with the Y direction as the projection of the unit's position onto this line in an orthogonal direction and Z as their mutual perpendicular. A consequence of this is that flexing of the chain does not preserve the end-point distances between consecutive cylinders or ellipsoids along the chain.

The length of cylinders and ellipsoids is set by reading in two end-points from the coordinate input data which have been pre-calculated from the inertial axes of the point-set that comprises the current unit (say, a secondary structure element or a domain). As well as the length, the line linking these end-points specifies the axis that corresponds with the X direction in the internal reference frame. The two end-points are then set within the data-structure that defines each unit as two points equidistant from the central point along the X direction. While the length of a unit is determined by these end-points, this is different from the size of each unit which is set generically for every unit on a given level by a value specified in the parameter file that describes the model. For a sphere, this is the only value that is needed and specifies the radius. For a cylinder, it also specifies the radius which is the thickness of the tube. For an ellipsoid, the end-points specify the length along the X axis and the size parameter specifies the other two axes. Therefore all ellipsoids are radially symmetric around X, giving a progression from oblate (disc) through spherical to prolate (cigar). Ignoring scalene ellipsoids excludes only long flat discs which are not common shapes for secondary structures or domains.

1.6 Implementation

1.6.1 Time and memory allocation

The adoption of a common data structure for each node in the hierarchy can lead to the allocation of memory for variables that are seldom, if ever, used. For example; the data structure allows for a general shape type which includes the coordinates of the end-points for tubes and ellipsoids yet if the object is a sphere, which it commonly is on the most populated atomic level, then space is wasted. Fortunately, with a reasonable workstation or laptop, memory is seldom a limitation for the system and tests have been made using over a million allocated nodes.

With a parallel implementation (using threads), the time allocated to the different processes can present scheduling considerations. A simple solution was adopted in which the call-back loop of each process was interleaved with a sleep call which suspended the process for a fixed period of time (currently 0.1 sec.). Within each process, higher priority was allocated to branches in the hierarchy that were in an active state, such as undergoing collision or close to a component that had been selected as being of special interest (such as the myosin molecule in the example considered below).

1.6.2 Visualisation

Objects were visualised in a simple viewer with all levels except the atomic being rendered as transparent according to the shape they had been given. Objects in a chain were linked by a thin tube which for spheres ran along the centre-centre direction and so was always normal to the spherical surface. For cylinders and ellipsoids, the linker tube ran from a sphere placed on the end-points, which for cylinders had the same radius as the cylinder (producing sausage-like objects) and for ellipsoids, was only slightly larger than the linker tube. This provides a visual distinction between spheres and spherical ellipsoids.

2 Actin/Myosin Example Application

To illustrate how such a system can be implemented in more concrete terms, we take the example of myosin-V on an actin filament, described in the main paper, in which large directed changes are applied to a component of the system (myosin) to drive it from one defined (bound) state to another then back to the original state but bound to a different actin molecule along the actin filament. The system will be introduced in two parts: firstly, by describing how the model was set-up as a hierarchical data structure, then secondly, how the dynamics were introduced. The first part is done through data files that have been alluded to in the previous section, however, the second part requires direct run-time interaction with the simulation and this is implemented as a specific user-defined routine called the `driver` (Fig.1) which interacts only with the common data structure and executes as an independent parallel process.

2.1 Model Construction

We will introduce the data-structure from the top down, starting with file (`actmyo.run`) that specifies the two main components: a myosin-V dimer and the actin filament.

actmyo.run

```
PARAM myosin.model
PARAM actin.model
END
GROUP 2
MODEL 0
INPUT myosin.dimer.dat
MODEL 1
INPUT actin.linear.dat
```

The above run file directs the INPUT from two files for the myosin dimer (`myosin.dimer.dat`) and an actin filament (`actin.linear.dat`) that constitute two units (GROUPs) at the highest level. Each group is preceded by a specifica-

tion of the parameter set (MODEL) that they will use (0 and 1) which corresponds to the PARAMeter files: `myosin.model` and `actin.model`, respectively. These files consist of columns of numbers with each column specifying the values for the different parameters at each level in the hierarchy. The file `myosin.model` consists of seven columns:

myosin.model

```
   0,    0,    0,    3,    1,    2,    1
9999, 1000,  500,  140,   70,   24,    5
   0,  100,  100,   50,   20,   20,   11
   0,    0,    0,    1,    2,    6,    4
   0,    0,    0,    1,    3,    1,    1
   0,    0,    1,    1,    1,    1,    1
   0,    1,    1,    1,    1,    1,    0
   0,    0,    1,    5,    5,    5,    5
   0,    0,    0,    1,    0,    0,    3
```

where the first is the state of the 'world' and the following six define six levels in the hierarchy. For example; the top line specifies the shape associated with each level with: 1=sphere, 2=cylinder, 3=ellipsoid and 0 being a virtual sphere that is not rendered. The following two lines specify the size of each object and its bumping radius (both in arbitrary units). The equivalent lines for the `actin.model` file show a slightly different structure using different values:

actin.model (part)

```
   0,    0,    1,    3,    1,    2,    1
9999, 3000,  120,  120,   70,   24,    5
   0,  400,  100,    0,   70,   20,   11
             :
```

2.1.1 Myosin

Considering firstly the myosin model, the file `myosin.dimer.dat` contains another two GROUPs both specified by the file `myosin.dat`:

myosin.dimer.dat

```
GROUP 2
SPINY 180.0
TRANS   55.0 -250.0 -450.0
INPUT myosin.dat
TRANS  -55.0 -250.0 -450.0
INPUT myosin.dat
```

The first INPUT is preceded by two geometrical transforms in which the contents of the file are rotated 180° about the Y axis (SPINY) then translated (TRANS x y z). The second file is only translated but in the opposite direction along X producing two copies related by a twofold axis, as seen in the X-ray crystal structure (PDB code: 2DFS) [Liu *et al.*, 2006]. (Figure 2).

The file `myosin.dat` contains two components of the myosin molecule which are referred to as the "head" and the "tail" and are set up as two GROUPs in what is now the third level in the hierarch. From the parameter file `myosin.model` it can be seen (col:4) that these objects are the first to have a defined shape and will behave and be rendered as ellipsoids.

myosin.dat

```
GROUP 2
TRANS  -31.0 136.5 59.0
INPUT myosin.head.dat
INPUT myosin.tail.dat
```

The file `myosin.head.dat` specifies the structure of the myosin head-group which is the globular kinase domain of the protein while the file `myosin.tail.dat` specifies the extended alpha-helical tail with its associated calmodulin-like light chains. (Sometimes referred to as the lever-arm or the "leg"). Although these files contain coordinate data from the same protein structure (`1DGS`), they have been processed separately which put their centroid to the origin and a translation (TRANS) was applied to the head group to restore their proper relative positions.

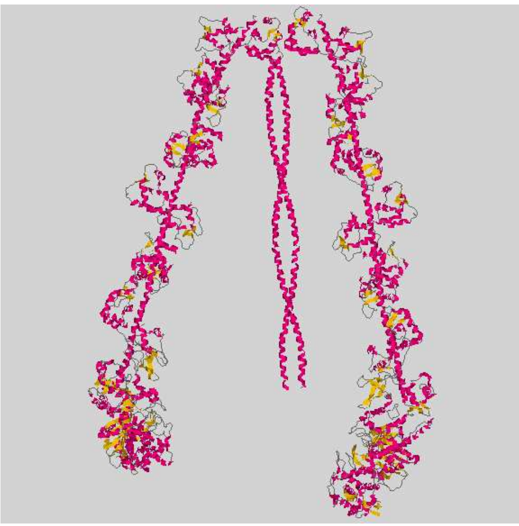

(a)

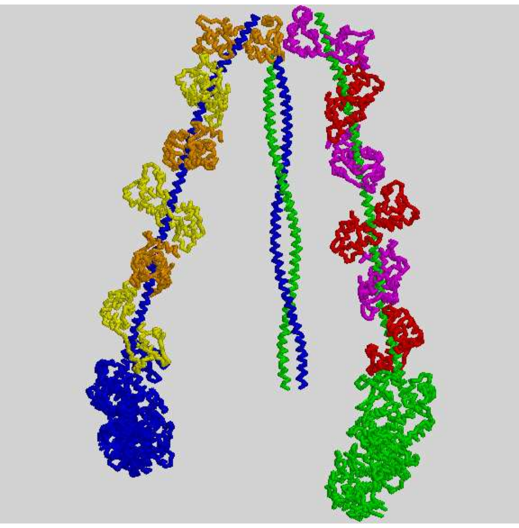

(b)

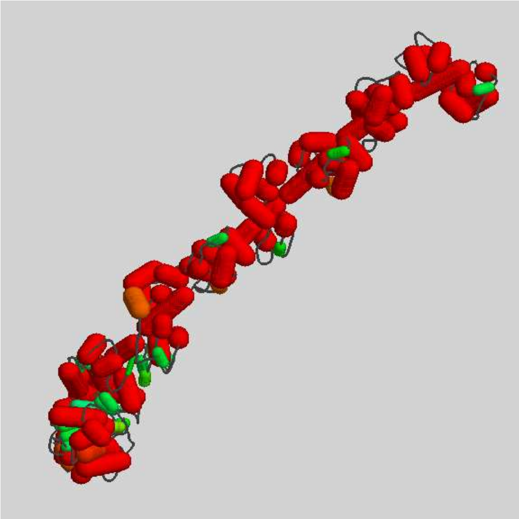

(c)

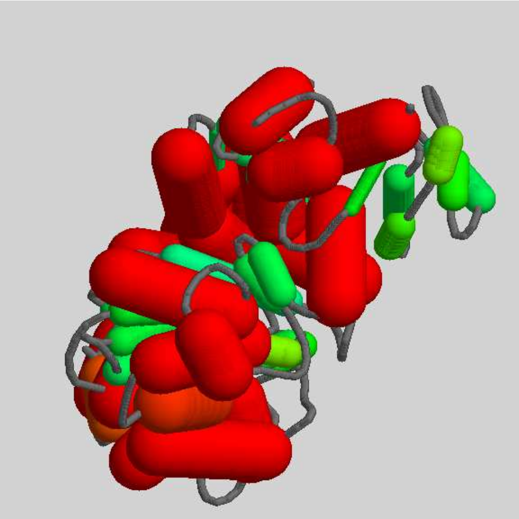

(d)

Figure 1: **Myosin-V structure.** The structure of dimeric myosin-V (`2DFS`), determined by single-particle cryo-electron microscopy is shown (*a*) with secondary structures represented in cartoon style (using RASMOL) with α-helix coloured pink and β-strands yellow. (*b*) The same structure is shown as a virtual α-carbon backbone with the two heavy chains coloured cyan and green and their associated light-chains in alternating yellow/orange and red/magenta, respectively. Secondary structure line-segments ("sticks") are shown as green tubes for β-strands and thicker red tubes for α-helices. (*c*) For the full structure of heavy and light chains (excluding the coiled-coil C-terminus) and (*d*) for the globular foot domain. (The amino-terminus lies in the all-β SH3 domain to the right)

By specifying the head and the tail as separate groups (units), they can be operated on independently by geometric transforms allowing the `driver` routine to recreate a large relative motion between them called the ”power-stroke” in which the tail swings through a large angle.

The myosin head group was split into seven domains as described previously () all of which are contained in distinct files: `head.dom[1-7].dat`. The level-3 unit that contains the domains was defined as an ellipsoid and its end-points are defined on the GROUP definition line (along with the number of children it contains).

myosin.head.dat

```
GROUP 7  -1.2 -42.6 -37.2    4.32 34.44 39.0
INPUT head.dom1.dat
INPUT head.dom2.dat
INPUT head.dom3.dat
INPUT head.dom4.dat
INPUT head.dom5.dat
INPUT head.dom6.dat
INPUT head.dom7.dat
REBOND 134 169
REBOND 275 320
REBOND 411 429
REBOND 495 412
REBOND 428 276
REBOND 292 541
REBOND 667 496
REBOND 540 293
REBOND 319 135
REBOND 168 668
```

The path of the chain through the seven head-group domains does not correspond simply to the domain order. Since each domain group is defined automatically by compactness, (giving sets of units that should move together), it is necessary to specify the chain path through the domains. This is done using the identity of units at the atomic level, sequentially numbered over the scope of the

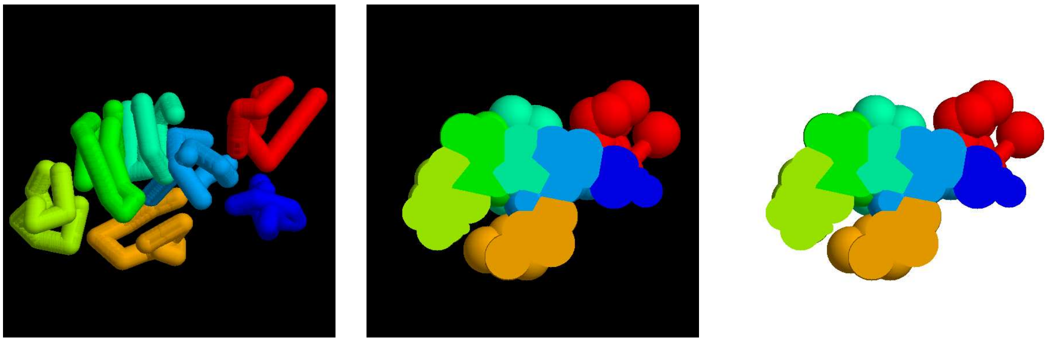

(a) (b) (c)

Figure 2: (*a*) SSEs allocated to produce seven domains: 3 core domains (light-blue, cyan, green), two binding domains (yellow, orange) and two ankle domains (blue, red). (*b*) The SSEs represented in Figure 2 are redrawn with space-filling spheres placed on each SSE end-point and sliced through by a plane that would also contain the 'leg' extension. (*c*) As in part *b* but with domain boundaries sketched and domain codes added: A1–2 (ankle), B1–2 (binding) and C1–3 (core). The direction of the X and Y axes of the internal reference frame are indicated by arrows.

current group. Each rewiring of the units is specified by a REBOND command which states that the unit with the first identity number should be bonded to the unit with the second identity number. For example; "REBOND 134 169" specifies that residue 134 should now link to residue 169. The resulting loose end at 135 is picked-up by the later command "REBOND 319 135". These connections can be specified "by-hand" but are written automatically by the preprocessing program that defines the domains.

Each domain file now takes us down to the lowest (atomic) level at which the actual X,Y,Z, coordinates are encountered. To avoid the proliferation of many small files, the two lowest levels (secondary structures and residues) are defined together with the first GROUP command stating that there are nine secondary structures in the group and the second (lower level) GROUP command specifying six ATOMS (atomic-level units) in each subgroup. The coordinate data (from the PDB file) is given at the atom level along with the secondary structure state encoded in the final column as: 1=loop, 2=beta and 3=alpha. As the secondary

structures are defined in the parameter file as cylinders, they are given end-point coordinates on the GROUP command line. If these are omitted, as with the first loop segment, default end-points are generated inside the program.

head.dom1.dat

```
GROUP 9
GROUP 6
ATOM      1  CA  GLY A   1      -3.537 -37.198 -20.771  1.00  1.00
ATOM      2  CA  GLY A   2      -1.470 -34.014 -20.628  1.00  1.00
ATOM      3  CA  GLY A   3       1.589 -35.231 -18.657  1.00  1.00
ATOM      4  CA  GLY A   4       3.165 -37.036 -21.663  1.00  1.00
ATOM      5  CA  GLY A   5       6.875 -37.568 -22.449  1.00  1.00
ATOM      6  CA  GLY A   6       8.601 -34.333 -23.626  1.00  1.00
GROUP 7     6.15 -31.57 -21.01   12.09 -24.98 -5.29
ATOM      7  CA  GLY A   7       6.079 -32.117 -21.857  1.00  2.00
ATOM      8  CA  GLY A   8       7.638 -29.779 -19.295  1.00  2.00
ATOM      9  CA  GLY A   9       6.751 -28.831 -15.716  1.00  2.00
ATOM     10  CA  GLY A  10       7.995 -26.626 -12.874  1.00  2.00
:
SHEET
BETA   44 36
BETA   43 37
BETA   37 43
BETA   36 44
BETA   36 23
BETA   23 36
```

The links between β-strands in a SHEET are specified as pairings on the BETA records at the end of the file.

The structure of the tail component follows along similar lines and will not be described in detail.

2.1.2 Actin

The highest level actin specific file, `actin.linear.dat` describes a segment of an actin filament which consists of repeated actin molecules related by helical symmetry with a rotation of -167° and 55Å translation. Because of the way they

interact with the myosin, actins were taken in pairs (called a dimer) giving a shift between dimers of 23° and 110Å[2]. These relationships could be encoded by separate rotate and translate commands but as helical symmetry is common, a combined HELIX command was created specifying the two components together:

——— actin.linear.dat ———

```
GROUP 16
INPUT actin.dimer.dat
HELIX 0.0 0.0 -5.8 26.0
INPUT actin.dimer.dat
HELIX 0.0 0.0 -11.6 52.0
INPUT actin.dimer.dat
HELIX 0.0 0.0 -17.4 78.0
INPUT actin.dimer.dat
HELIX 0.0 0.0 -23.2 104.0
INPUT actin.dimer.dat
:
```

Each `actin.dimer.dat` file simply introduces another level in the hierarchy and maintains the same symmetry around the fibre axis but because the dimer centre lies on the axis, only a shift along Z is needed at the higher level.

——— actin.dimer.dat ———

```
GROUP 2
HELIX  -3.55  -0.45  0.0    0.0
INPUT actin.one.dat
HELIX   3.55   0.45 -2.9 -167.0
INPUT actin.one.dat
```

The actin molecule consists of four domains forming a flat disc which was naturally encoded as an oblate ellipsoid. This was (hand) specified by an axis of length 10 (-5 to 5) which relative to the size of the domain specified in `actin.model` gives an excessive axial ratio which is set to the maximum allowed ratio of 5.

——— actin.one.dat ———

[2]NB: the internal coordinates in the data structure are not Ångstroms

```
GROUP 4    -5.0  0.0  0.0    5.0  0.0  0.0
INPUT actin.dom1.dat
INPUT actin.dom2.dat
INPUT actin.dom3.dat
INPUT actin.dom4.dat
RELINK 32 127
RELINK 172 33
RELINK 90 173
RELINK 216 281
RELINK 372 217
RELINK 280 91
ENDS   372 126
```

As with the myosin head domains, the chain path through the actin domains must be relinked. This also entails the creation of a new terminal position which is specified by the ENDS command. (The equivalent myosin command falls in the tail segment).

Each domain file (`actin.dom[1-4].dat`) is similar to the equivalent myosin head group domain files and introduce no novel features.

2.2 Driver construction

The `driver` routine encodes the dynamic aspect of the myosin motor. This includes specifying the actin/myosin bound state along with motion at the hinge points between the myosin head and tail and the myosin dimer. From any given starting configuration, the actin lying closest to the myosin head was identified and the myosin molecule moved towards it. When the myosin came within a predefined distance, its orientation was also refined. Together these operations "docked" the myosin molecule into a configuration relative to the actin that corresponded to the known structure. When in this position (referred to as “tightly bound”), the orientation of the myosin tail relative to the head was rotated about an axis that corresponded to what can be inferred from the structures of myosin with tails in different positions. This motion, known as the "power-stroke" can

only occur if the other half of the myosin dimer is unbound. In this situation, the free myosin will be carried along towards a new region of the actin filament where it will then search for a new binding site. Cycling through these states leads to a processive motion of myosin along the actin filament[3].

The mechanics of the myosin walking motion can be decomposed into three distinct components: the myosin can be bound or unbound to actin, the head can be either in a pre- or post- power-stroke position and the two halves of the myosin dimer can swivel around their dimer interface. There is no explicit communication between the binding states of the two myosin molecules, except what can be communicated through their dimer interface. This has the form of an alpha-helical coiled-coil but little is known of how it responds under stress (tension) or how it affects the diffusion search of an unbound head. By contrast, the power-stroke (PS) transition is well characterised by many structural studies and the forward stroke (from pre- to post-PS positions) occurs only when the myosin is bound to actin whereas the reverse stroke (cocking the trigger) occurs in the unbound state.

2.2.1 The myosin dimer hinge

The most independent component of the myosin machine is the dimeric interface which, by analogy to walking motion, will be referred to as the 'hip' joint. As little is known about its' structure or dynamics, it was modelled simply as a constraint to hold the ends of the legs at a fixed distance.

Walking motion requires movement of the legs about the hip-joint and rather than rely on the generic diffusion built into the geometry engine, an additional motion was included in the `driver` routine to give any unbound myosin a rotational displacement about the hip. This provides an example of how the `driver`

[3]Note that this processive, or proper walking, motion of myosin-V differs from that of muscle myosin (II) in which the actin myosin contact is only transient. The latter is more akin to a bank of rowers in which each myosin oar is dipped into the actin river.

routine can utilise structural information across levels in the hierarchy as the rotation is applied to the whole myosin molecule (level-2) whereas the axis is determined by the domain positions at the end of the tail in both molecules (level-4 in separate sub-trees).

While a faster rate of driven motion reduces the search time for the free myosin head to find a new binding site without disrupting the position of the bound head, it also gives less time for the generic collision detection algorithm to avert clashes between the myosin molecules and the actin filament. To rectify this, a specific high-level check was made on the moving myosin based on the distance of the head from the actin filament (the actin dimer centre) and the other head group. The tail was also checked but as this is an elongated substructure, the closest approach of the ellipsoid major axes was monitored and if these fell below a fixed cutoff (set at the same distance as the hip joint) then the two myosins were separated.

2.2.2 The actin/myosin binding

The distance of the myosin head from each actin dimer centre was monitored and when this fell within a given range, the myosin was moved closer to the selected actin. This choice is made afresh every time the `driver` routine is activated, so the target actin can vary as the configuration of molecules changes. This approach mode, referred to as "loose binding", which includes no orientation component, continues until a shorter threshold is reached at which point the closer actin molecule is selected and the myosin is orientated and translated towards its known binding position. The orientation component is calculated based on the internal reference frames of the actin and the myosin head by applying the rotation matrix and translation vector that reproduces the docked complex. A fully-bound state is declared if this transformation can be applied and the end of the tail is placed within the range of the hip-joint to the other myosin.

2.2.3 The power-stroke

The power-stroke (PS) consists of a large swinging motion of the tail relative to the head which is easily encoded in the `driver` routine as a rotation about an axis that lies within the head. Structural studies have associated the swivel point with a particular α-helix and the coordinates of this helix centre were taken as the hub. The rotation axis is less well defined but can also be inferred from the known structures and this was calculated and set as a fixed axis in the `driver` routine. Similarly, the extent of the left and right swing were preset with reference to the internal reference frame of the myosin head.

Given these constraints, a simple mechanism was encoded that maintained a position at the end of the swing range depending on the bound state of the myosin. If the myosin was unbound the tail angle was incremented until it attained the pre-PS position and only when the myosin became fully bound, was this motion reversed towards the post-PS position. These two states were used to impose an additional condition on binding: that the myosin can only select an actin for binding when it is in the pre-PS position. This means that the post-PS state must detach from the actin and revert to the pre-PS position before it can rebind.

References

[Katsimitsoulia & Taylor, 2010] Katsimitsoulia, Z. & Taylor, W. R. (2010). A hierarchic algorithm for simple Brownian dynamics. *Compu. Biol. Chem,* **34**, 71–79. doi.10.1016/j.compbiolchem.2010.01.001.

[Liu *et al.*, 2006] Liu, J., Taylor, D. W., Krementsova, E. B., Trybus, K. M. & Taylor, K. A. (2006). Three-dimensional structure of the myosin v inhibited state by cryoelectron tomography. *Nature,* **442**, 208–211.

[Taylor & Katsimitsoulia, 2010] Taylor, W. R. & Katsimitsoulia, Z. (2010). A soft collision detection algorithm for simple Brownian dynamics. *Compu. Biol. Chem,* **34**, 1–10. doi.10.1016/jcompbiolchem.2009.11.003.
